# Are people with amyotrophic lateral sclerosis (ALS) particularly nice? An international online case–control study of the Big Five personality factors

**DOI:** 10.1002/brb3.1119

**Published:** 2018-09-21

**Authors:** Jane A. Parkin Kullmann, Susan Hayes, Roger Pamphlett

**Affiliations:** ^1^ The Stacey Motor Neuron Disease Laboratory, Discipline of Pathology The University of Sydney Sydney New South Wales Australia; ^2^ Forensic Psychology, Sydney Medical School The University of Sydney Sydney New South Wales Australia; ^3^ Department of Neuropathology Royal Prince Alfred Hospital Sydney Sydney New South Wales Australia

**Keywords:** amyotrophic lateral sclerosis, Big Five Inventory, case‐control study, international, online questionnaire, personality, risk factors

## Abstract

**Background:**

Many people with ALS have been suggested to have a “nice” personality, but most ALS personality studies to date have had limited numbers of participants and have not taken into account personality differences between genders. We used Big Five Inventory data obtained from an online questionnaire looking for risk factors for ALS to investigate personality traits in large numbers of people with ALS and controls.

**Methods:**

A total of 741 questionnaire respondents aged 40 years and over indicated the extent to which they agreed with each of the 44 Big Five Inventory statements. Respondents were 339 with ALS (212 male, 127 female) who responded to the statements as they applied to them before their diagnosis and 402 controls (120 male, 282 female). Unpaired *t* tests with 95% confidence intervals were used to compare mean values of Big Five‐factor scores.

**Results:**

Female respondents taken together had higher mean scores for Agreeableness and Neuroticism than all male respondents. Male ALS respondents had higher mean scores than male controls for Conscientiousness and Extraversion. Female ALS respondents had higher mean scores than female controls for Agreeableness, Conscientiousness, and Extraversion, and a lower score for Neuroticism.

**Conclusions:**

Many people with ALS have personality traits that are likely to underlie the perception they are particularly “nice.” This raises the possibility that genetic polymorphisms that influence personality could play a role in ALS. Furthermore, different personality traits could underlie lifestyle choices that are currently thought to be risk factors for ALS.

## 1 INTRODUCTION

People with amyotrophic lateral sclerosis (ALS), also known as motor neuron disease, are often described by clinicians as having a particularly pleasant personality, which could be interpreted as “niceness” (Mehl, Jordan, & Zierz, 2017). Interest has persisted in this subject because of the possibility that a characteristic personality profile for ALS could give clues as to the underlying pathogenesis of the disease (Brown & Mueller, 1970; Grossman, Levin, & Bradley, 2006; Mehl et al., 2017).

The previous studies of personality differences in ALS go back to 1947 when ALS patients were reported to have a “cheerful” attitude (Veit, 1947). In 1970, a range of psychological tests characterized 10 ALS patients as having “active mastery” and “denial of affect,” with the conclusion that an “association of ALS with a characteristic personality style, if confirmed, might have etiologic and prognostic implications” (Brown & Mueller, 1970). In 1977, a study of 40 ALS patients, using a variety of psychological tests, was unable to find any of the denial of affect (Houpt, Gould, & Norris, 1977) that had been reported in the 1970 study. It was suggested that small numbers, and issues with patient selection, were the reasons for this discrepancy. The next ALS personality study in 1978 found no differences in Minnesota Multiphasic Personality Inventory profiles of 38 ALS patients compared with a large number of medical patients without serious conditions (Peters, Swenson, & Mulder, 1978).

A different approach was taken in 2006 when 49 caregivers were asked to rate the premorbid personality traits of ALS patients, compared to non‐ALS patients with chronic progressive conditions, using the NEO Five‐Factor Personality Inventory (Grossman et al., 2006). ALS patients were rated as having comparatively lower openness, but the other factors of neuroticism, extraversion, agreeableness, and conscientiousness did not differ between the two groups.

When 36 physicians assessed the personality of their ALS patients in a 2017 study, using a shortened version of the NEO Five‐Factor Personality Inventory, agreeableness was rated more highly than that reported by physicians caring for patients with non‐ALS illnesses (Mehl et al., 2017). This supported the anecdotal description of people with ALS as being “nice.”

In summary, ALS premorbid personality studies have to date given mixed results. No systematic studies of premorbid personality in large numbers of people with ALS have been reported, and none has taken into account gender differences in personality. In an attempt to find out if certain personality types are more common in people with ALS, including those associated with “niceness,” we used personality data generated by the Big Five Inventory (John, Naumann, & Soto, 2008) from ALS Quest, an online international questionnaire designed to study risk factors for ALS (Parkin Kullmann, Hayes, Wang, & Pamphlett, 2015). The large numbers of ALS and control respondents to this questionnaire enabled the study of personality factors in men and women separately.

## 2 METHODS

### 2.1 Setting

This case–control study used data collected between January 2015 and February 2017 from a multilingual web‐based questionnaire, ALS Quest (http://www.alsquest.org) (Parkin Kullmann & Pamphlett, 2017; Parkin Kullmann et al., 2015). Respondents were recruited via worldwide national and state ALS Associations, national ALS registries, the Internet, and social media. No personally identifying data were collected so respondents remained anonymous. Information on disease status was self‐reported. Cases were respondents who stated “Yes, I have been diagnosed with ALS/MND.” Controls were participants who stated “No, I have not been diagnosed with ALS/MND.”

### 2.2 Ethics, consent, and permissions

The project was approved by the Human Ethics Committee of the Sydney Local Health District. Participants consented to submit their questionnaire responses by clicking an “I consent” button after acknowledging that they had read the preceding Information for Participants section of the questionnaire and agreeing that they “Understand that I will not be asked for any personal information that could identify me, so the study is anonymous and strictly confidential” and “Freely choose to participate in the study and understand that I can withdraw my questionnaire answers at any time until I click the Submit button at the end of the questionnaire.”

### 2.3 The Big Five Inventory

The Big Five Inventory provides scores for the personality factors of Agreeableness, Conscientiousness, Extraversion, Neuroticism, and Openness (John et al., 2008). These are calculated from a set of 44 statements about personality. Each statement is rated on a five‐point Likert scale ranging from strongly disagree to strongly agree. Descriptors for these factors by the Big Five Inventory developers are as follows: (a) Agreeableness: altruism, tender mindedness, trust, and modesty; (b) Conscientiousness: thinking before acting, delaying gratification, following norms and rules, and planning, organizing, and prioritizing tasks; (c) Extraversion: sociability, activity, assertiveness, and positive emotionality; (d) Neuroticism: feeling anxious, nervous, sad, and tense; and (e) Openness: the breadth, depth, originality, and complexity of a person's mental and experiential life. The Big Five Inventory statements were translated into the language used by the respondent to complete the questionnaire. People with ALS were asked to provide responses they would have given to the statements *before* their diagnosis of ALS was made.

### 2.4 Exclusion criteria

**Figure 1:**
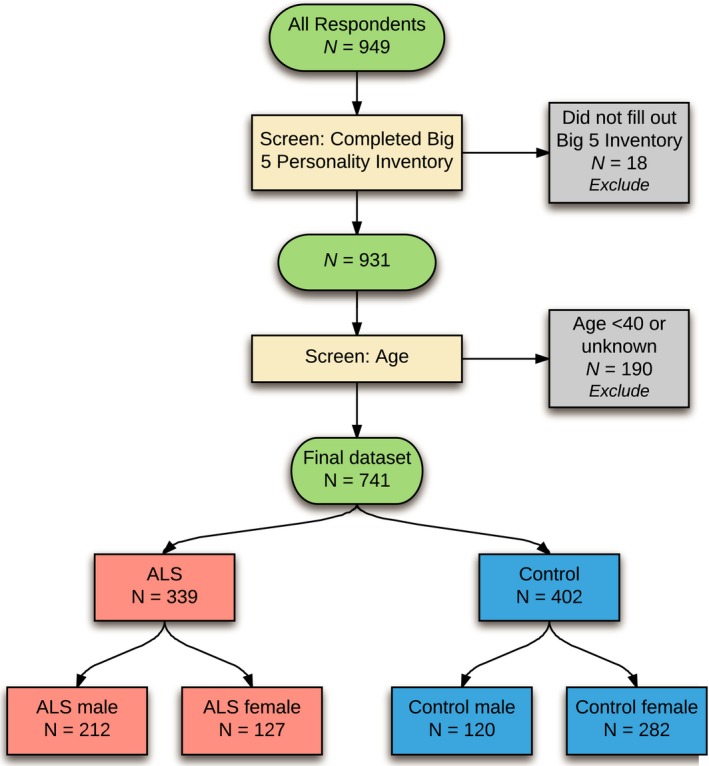
Selection of individuals for analysis. The flowchart shows the final dataset of 741 respondents after exclusion criteria were applied and final numbers of ALS and control individuals

Respondents were excluded from analysis (Figure 1) if they did not complete the Big Five Inventory, they did not supply their age, or they were under the age of 40 years. The latter exclusion was to limit the number of control respondents who might later develop ALS and to avoid differences in personality between younger and older individuals (Srivastava, John, Gosling, & Potter, 2003).

### 2.5 Big five‐factor scores

A mean score, ranging from 1 to 5, was calculated for each factor, with higher scores indicating a greater expression of the factor. Percentage frequency histograms were created to compare the distributions of scores between ALS respondents and controls.

### 2.6 Clinical characteristics

People with ALS were asked to complete the online version of the ALS Functional Rating Scale‐Revised that has been designed for self‐assessment (Maier et al., 2012; Parkin Kullmann et al., 2015) to assess their physical state at the time of taking the questionnaire; scores were inverted from the standard scale so that the higher the score the more impaired the function, with 0 indicating no impairment and 48 indicating very severe impairment. This online self‐assessment of ALS severity has been shown to have excellent agreement with face‐to‐face application of the rating scale (Maier et al., 2012). The disease duration at the time of completing the questionnaire was calculated by subtracting the year of disease onset from the year of consenting to complete the questionnaire.

### 2.7 Data analysis

Data from the Qualtrics server were transferred to GraphPad Prism 7 files. *F* tests were used to compare variances between groups, before unpaired *t* tests with 95% confidence intervals were used to compare mean values of Big Five scores. In only two groups (ALS male vs. female for Agreeableness and female ALS vs. control for Openness) were slight differences in variances found, but when these analyses were repeated using nonparametric Mann–Whitney tests, the same results were found. Correlations between the personality scores and the ALSFRS‐R inverted scale and disease duration were measured with Spearman nonparametric r coefficients. Effect sizes (*d*) were calculated using G*Power software. Significance was assessed at the 0.05 level.

Lucidchart software was used to create Figures 1 and 7.

## 3 RESULTS

### 3.1 Cases and controls

A total of 741 respondents were eligible for analysis after exclusion criteria were applied (Figure 1). These comprised 339 ALS respondents (212 male and 127 female) and 402 non‐ALS controls (120 male and 282 female). The mean age of ALS respondents was 61.3 years (*SD*: 9.3 years, range: 40–84 years) and of controls was 57.3 years (*SD*: 10.6 years, range: 40–89 years). Information on countries of birth and residence, ancestries, and cultural groups of respondents can be viewed in Table 1. The composition of the ALS and control groups was similar with regard to country of birth, country of residence, ancestry, and cultural group. The majority of respondents resided in Australia, the USA, and Canada, though residents of a further 27 countries supplied responses.

**Table 1:** Demographic characteristics of respondents

	ALS	*n* (%)	CONTROL	*n* (%)
Country of birth	United States	128 (38)	Australia	248 (62)
	Australia	83 (25)	Other (<2% each)	67 (17)
	Canada	48 (14)	United States	38 (10)
	Other (<1% each)	52 (15)	United Kingdom	24 (6)
	United Kingdom	17 (5)	Spain	13 (3)
	Spain	10 (3)	South Africa	10 (3)
Country of residence	United States	136 (40)	Australia	305 (76)
	Australia	103 (34)	United States	44 (11)
	Canada	56 (17)	Other* (<1% each)	28 (7)
	Other* (<1% each)	34 (10)	Spain	13 (3)
	Spain	9 (3)	Canada	5 (1)
			New Zealand	5 (1)
Ancestry	Other (<5% each)	162 (49)	Other (<4% each)	138 (35)
	Australian	51 (15)	Australian	115 (29)
	English	51 (15)	English	69 (17)
	American	27 (8)	Irish	37 (9)
	Irish	21 (6)	British	22 (6)
	German	21 (6)	Scottish	16 (4)
Cultural group	Australian	87 (27)	Australian	255 (64)
	American	81 (25)	Other (<2% each)	65 (16)
	Other (<2% each)	79 (24)	American	29 (7)
	Canadian	39 (12)	English	27 (7)
	English	28 (9)	Spanish	13 (3)
	Spanish	10 (3)	Dutch	8 (2)

Note

Other* countries of residence: Argentina, Belgium, Brazil, China, Colombia, Denmark, Ecuador, Egypt, Finland, Germany, Iran, Ireland, Italy, Luxembourg, Mexico, Netherlands, Portugal, Russia, Slovakia, South Africa, South Korea, Sweden, Switzerland, Turkey, and the United Kingdom.

ALS: amyotrophic lateral sclerosis.

Sources of information about the questionnaire cited by respondents were as follows: ALS Associations (36%), the Internet (22%), friends (9%), people with ALS (7%), health professionals (6%), community groups (5%), Facebook (4%), the USA CDC National ALS Registry (3%), the Canadian Neuromuscular Disease Registry (2%), and ALS researchers (2%).

About 9% of ALS respondents had at least one relative who had been diagnosed with ALS and were classified as having familial ALS. The other 91% of ALS respondents were classified as having sporadic (or isolated) ALS. As reported by the ALS respondents, 58% had “classic” (upper and lower motor neuron variant) ALS, 9% progressive muscular atrophy (lower motor neuron variant), 8% progressive bulbar palsy, 8% primary lateral sclerosis (upper motor variant), 7% “other,” 2% ALS/FTD (frontotemporal dementia), and 8% did not know their subtype of ALS.

When control participants were asked what their connection with ALS was, responses were (blood and nonblood) relatives (45%), spouses (10%), or friends (13%) of people with ALS, individuals from community, research, or medical professional groups (9%), or no specific (or another type of) connection to ALS/MND (23%). When controls were asked to identify what blood relatives with ALS they had, 58% reported no blood relatives with ALS, whereas 29% had one, and 13% more than one, blood relative/s with ALS.

### 3.2 Big Five**‐**factor mean scores

#### 3.2.1 Male versus female differences

**Figure 2:**
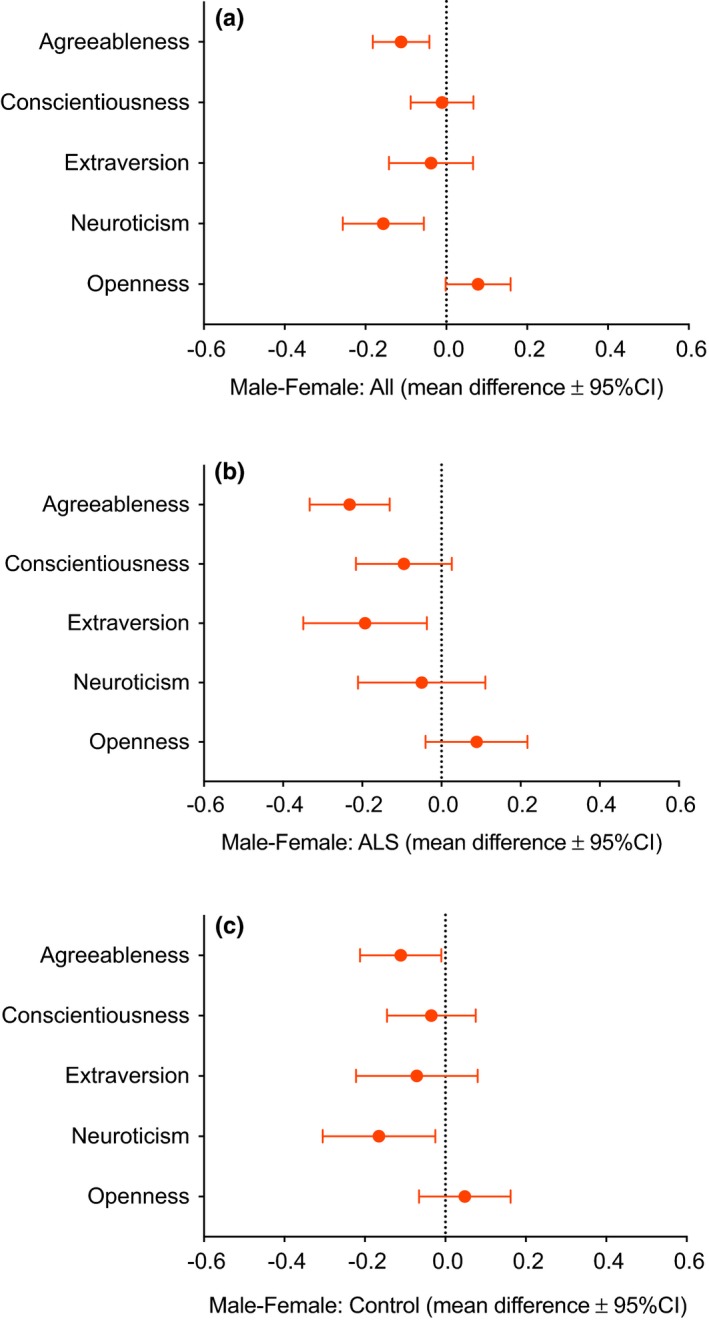
Mean differences (filled circles) and 95% confidence intervals (bars) in Big Five factors in males and females. Graphs for (a) males and female combined, (b) ALS males and females, and (c) control males and females show the differences in mean scores between groups. For example, in (a), it can be seen that women overall scored significantly more (i.e., the 95% CI bars do not cross the dotted zero line) for Agreeableness than men, while men tended to score more for Openness

When all men and all women were compared, women scored higher than men for Agreeableness and Neuroticism (Figure 2a). No significant differences were seen between men and women for Extraversion, Conscientiousness, or Openness.

ALS females scored higher than ALS males for Agreeableness (effect size *d* = 0.49) and Extraversion (*d* = 0.27) (Figure 2b, Table 2). No significant differences were found between these two groups for Conscientiousness, Openness, or Neuroticism.

**Table 2:** Differences in Big Five‐factor scores between male (control *n* = 120, ALS *n* = 212) and female (control *n* = 282, ALS *n* = 127) respondents

Big Five factors	Group	Big Five‐factor score mean (*SD*)	Big Five‐factor score mean difference (95% CI)	*p* Value	Effect size (*d*)
1. Agreeableness	Male control	3.71 (0.51)	−0.11 (−0.21 to −0.01)	0.03	0.23
	Female control	3.83 (0.46)			
	Male ALS	3.83 (0.51)	−0.23 (−0.34 to −0.13)	<0.001	0.49
	Female ALS	4.06 (0.43)			
2. Conscientiousness	Male control	3.84 (0.52)	−0.04 (−0.15 to 0.08)	0.54	
	Female control	3.88 (0.51)			
	Male ALS	3.97 (0.56)	−0.10 (−0.22 to 0.03)	0.12	
	Female ALS	4.07 (0.53)			
3. Extraversion	Male control	3.10 (0.70)	−0.07 (−0.22 to 0.08)	0.36	
	Female control	3.17 (0.71)			
	Male ALS	3.32 (0.70)	−0.19 (−0.35 to −0.04)	0.02	0.27
	Female ALS	3.51 (0.71)			
4. Neuroticism	Male control	2.60 (0.63)	−0.17 (−0.31 to −0.03)	0.02	0.26
	Female control	2.77 (0.64)			
	Male ALS	2.52 (0.75)	−0.05 (−0.21 to 0.11)	0.54	
	Female ALS	2.57 (0.69)			
5. Openness	Male control	3.52 (0.54)	0.05 (−0.07 to 0.16)	0.40	
	Female control	3.47 (0.53)			
	Male ALS	3.57 (0.56)	0.09 (−0.04 to 0.22)	0.18	
	Female ALS	3.48 (0.62)			

Note

ALS: amyotrophic lateral sclerosis; *SD*: standard deviation; CI: confidence interval.

Control females scored higher than control males for Agreeableness (*d* = 0.23) and Neuroticism (*d* = 0.26) (Figure 2c, Table 2). No significant differences were found between these two groups for Conscientiousness, Openness, or Extraversion.

#### 3.2.2 ALS versus control differences

**Figure 3:**
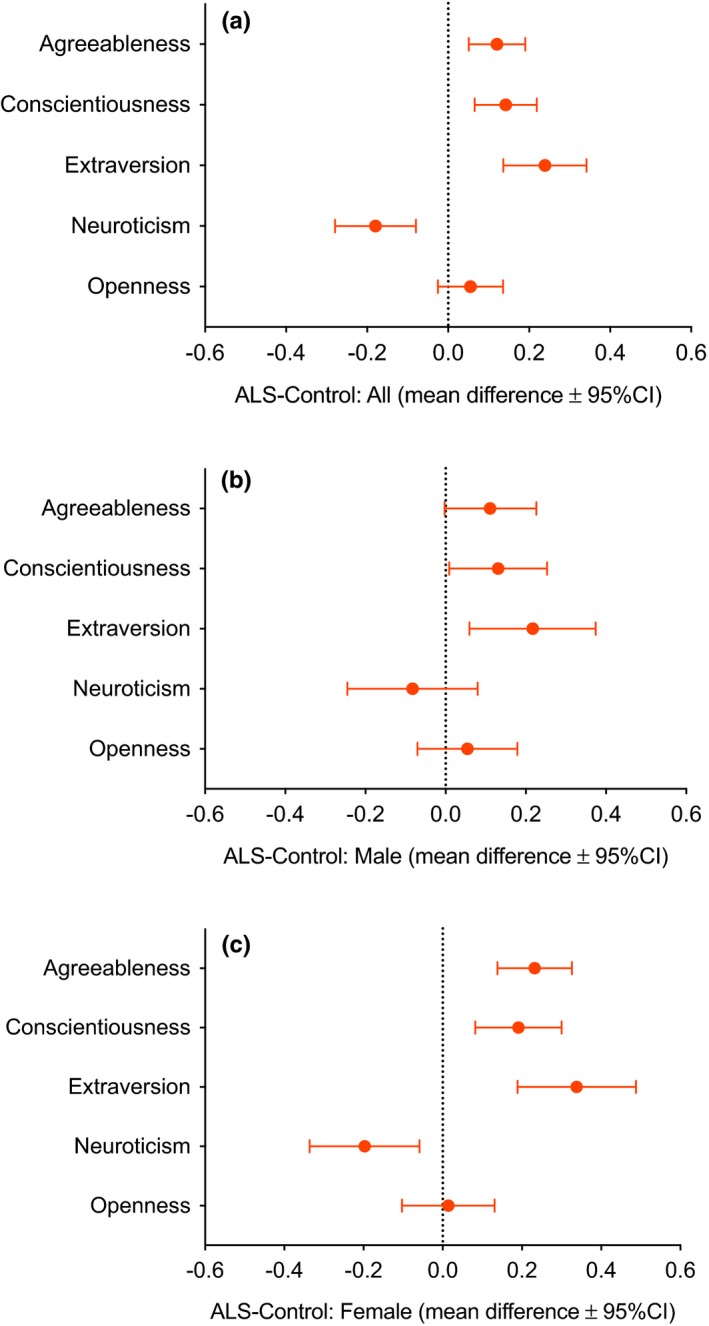
Mean differences (filled circles) and 95% confidence intervals (bars) in Big Five factors in ALS respondents and controls. Graphs for (a) males and female combined, (b) ALS males and controls, and (c) ALS females and controls show differences in mean scores. For example, in (a), it can be seen that ALS respondents overall scored significantly more for Agreeableness, Conscientiousness, and Extraversion, less for Neuroticism, and similarly for Openness

When all ALS respondents were compared to all controls, ALS respondents scored higher than controls for Agreeableness, Conscientiousness, and Extraversion, and lower than controls for Neuroticism (Figure 3a, Table 3). No significant difference was seen in Openness.

**Table 3:** Differences in Big Five‐factor scores between ALS (male *n* = 212, female *n* = 127) and control (male *n* = 120, female *n* = 282) respondents

**Big Five factors**	**Group**	**Big Five‐factor score mean (*SD*)**	**Big Five‐factor score mean difference (95% CI)**	***p* Value**	**Effect size (*d*)**
1. Agreeableness	Male control	3.71 (0.50)	−0.11 (−0.23 to 0.002)	0.06	
	Male ALS	3.83 (0.51)			
	Female control	3.83 (0.46)	−0.23 (−0.33 to −0.14)	<0.001	0.54
	Female ALS	4.06 (0.43)			
2. Conscientiousness	Male control	3.84 (0.52)	−0.13 (−0.25 to −0.009)	0.04	0.24
	Male ALS	3.97 (0.56)			
	Female control	3.88 (0.51)	−0.19 (−0.30 to −0.08)	0.001	0.38
	Female ALS	4.07 (0.53)			
3. Extraversion	Male control	3.10 (0.70)	−0.22 (−0.37 to −0.06)	0.007	0.31
	Male ALS	3.32 (0.70)			
	Female control	3.17 (0.71)	−0.34 (−0.49 to −0.19)	<0.001	0.48
	Female ALS	3.51 (0.71)			
4. Neuroticism	Male control	2.60 (0.67)	0.08 (−0.08 to 0.25)	0.32	
	Male ALS	2.52 (0.75)			
	Female control	2.77 (0.64)	0.20 (0.06 to 0.34)	0.005	0.30
	Female ALS	2.57 (0.69)			
5. Openness	Male control	3.52 (0.54)	−0.05 (−0.18 to 0.07)	0.39	
	Male ALS	3.57 (0.56)			
	Female control	3.47 (0.53)	−0.01 (−0.13 to 0.10)	0.82	
	Female ALS	3.48 (0.62)			

Note

ALS: amyotrophic lateral sclerosis; *SD*: standard deviation; CI: confidence interval.

Male ALS respondents scored higher than male controls for Extraversion (*d* = 0.31) and Conscientiousness (*d* = 0.24) (Figure 3b, Table 3). No significant differences were found for Openness, Neuroticism, or Agreeableness.

Female ALS respondents scored higher than female controls for Agreeableness (*d* = 0.54), Conscientiousness (*d* = 0.24), and Extraversion (*d* = 0.48), and lower than controls for Neuroticism (*d* = 0.30) (Figure 3c, Table 3). No significant differences were found for Openness.

No significant differences in mean scores were seen when the 91% of sporadic ALS respondents were compared with the 9% of familial ALS respondents (data not shown).

### 3.3 Correlation of Big Five‐factor scores with ALS functional state and duration

**Figure 4:**
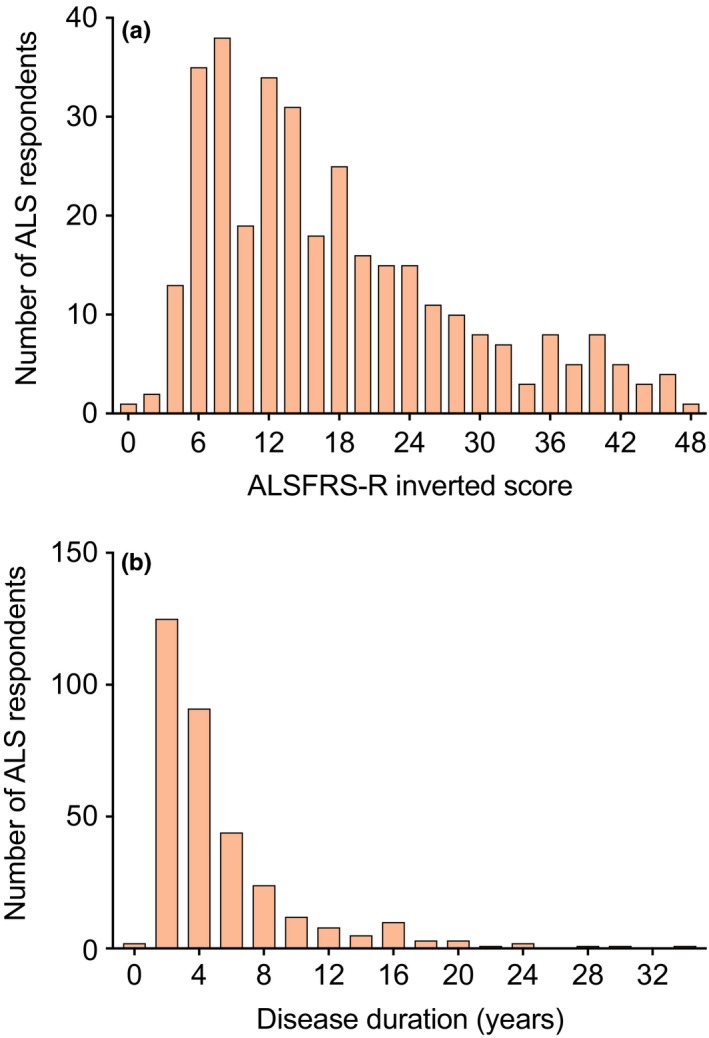
ALS functional state and duration. (a) Most ALS respondents were in the mild or moderate functional impairment range (on the left side of the histogram) as measured by the ALS Functional Rating Scale (ALSFRS‐R) inverted score. A few respondents (on the right side of the histogram) were severely affected. (b) The majority of ALS respondents replied to the questionnaire within the first four years after disease onset (on the left side of the histogram), though a few long‐term survivors also completed the questionnaire (on the right side of the histogram)

Most ALS respondents had only mild or moderate loss of function as measured by the ALSFRS‐R inverted score (Figure 4a); the median ALSFRS‐R inverted score was 14. Most responded within the first four years after their ALS disease onset, with a long tail containing a few long‐term survivors (Figure 4b); the median disease duration was 3 years. Correlation coefficients between the personality factors of Agreeableness, Conscientiousness, Extraversion, Neuroticism, Openness, and the ALSFRS‐S inverted scores were low (respectively, *r* = −0.03, 0.06, 0.02, 0.02, and 0.08, all nonsignificant), with similar low personality score correlations with disease duration (respectively, *r* = 0.06, 0.00, 0.10, 0.01, and 0.03, all nonsignificant). The ALSFRS‐R inverted score correlated significantly with disease duration (*r* = 0.31, *p* < 0.001).

### 3.4 *Big Five‐factor frequency distributions: ALS* versus* controls*

**Figure 5:**
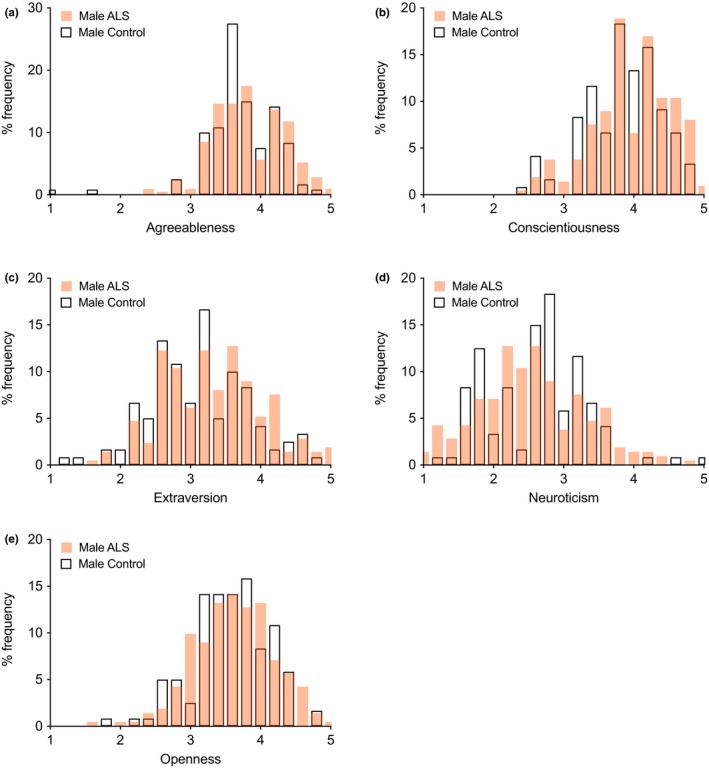
Percentage frequency distributions of male ALS and control individuals. The two distributions overlap, but male ALS distributions are shifted to the right for Agreeableness, Conscientiousness, and Extraversion, and slightly to the left for Neuroticism

**Figure 6:**
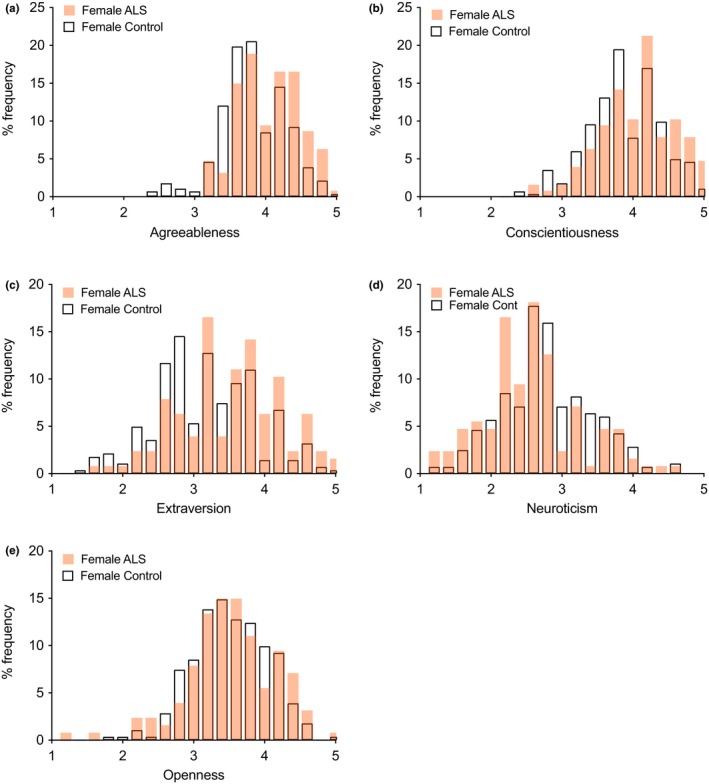
Percentage frequency distributions of female ALS and control individuals. The two distributions overlap, but female ALS distributions are shifted to the right for Agreeableness, Conscientiousness, and Extraversion, and to the left for Neuroticism

Frequency percentage histograms showed overlap in Big Five scores between ALS respondents and controls, both for men (Figure 5) and women (Figure 6), but with shifts in some ALS distributions to the right or left in line with the mean score results.

**Figure 7:**
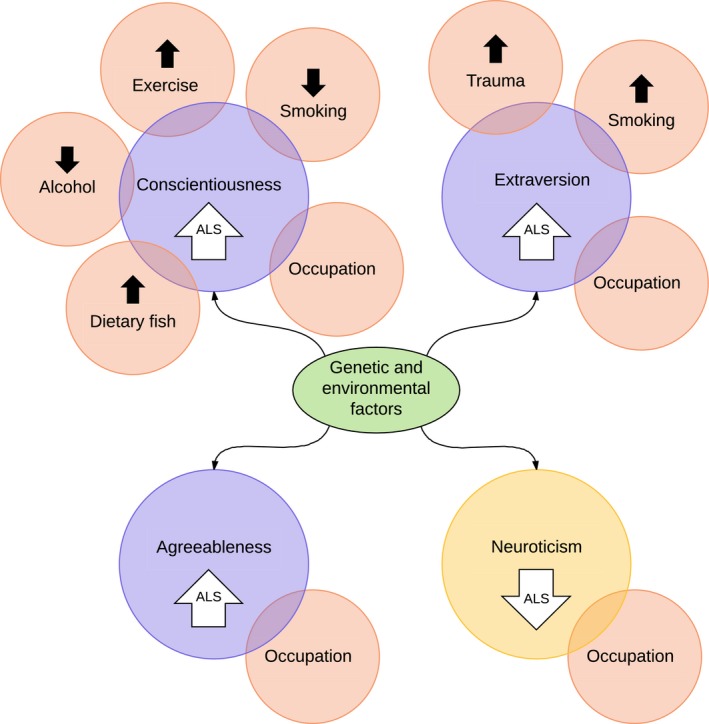
Potential interactions between the four ALS‐related personality traits described in the present study (large circles) and postulated ALS risk factors (small circles). Both genetic and environmental factors underlie personality traits. Increased Conscientiousness in ALS could be associated with increased exercise, decreased alcohol intake, and increased fish consumption, all possible ALS risk factors. Increased Extraversion could be associated with increased smoking and increased risk‐taking behavior leading to head trauma, both reported ALS risk factors. All four personality factors could influence choices of occupations, with some occupations being associated with the risk of ALS

## 4 DISCUSSION

We sought first to find out whether personality differences existed between our men and women respondents. Women scored higher than men for Agreeableness and Neuroticism, as has been reported in other studies (Weisberg, Deyoung, & Hirsh, 2011), so to compare ALS and control individuals, we analyzed male and female respondents separately. The mean Big Five scores for ALS males were higher for Extraversion and Conscientiousness. For ALS females, the scores were higher for Agreeableness, Conscientiousness, and Extraversion, and lower for Neuroticism. These particular ALS traits taken together would accord with many people's concept of what constitutes “niceness.” For example, the Oxford English Dictionary provides the following synonyms for “nice” when referring to a person: “pleasant, likeable, agreeable, personable, charming, delightful, amiable, affable, friendly, kindly, genial, congenial, good‐natured, engaging, gracious, sympathetic, understanding, compassionate, good” (https://en.oxforddictionaries.com/definition/nice). Several descriptors in this list could be applied to our findings in ALS of increased Agreeableness, Extraversion, and Conscientiousness, as well as decreased Neuroticism. These personality traits could therefore underlie the widespread perception, in particular among treating physicians, that people with ALS are unusually pleasant, even in the face of a devastating disease (Mehl et al., 2017).

None of the Big Five scores for ALS respondents correlated significantly with the ALS Functional Rating Scale scores, indicating that disease severity at the time of taking the questionnaire did not influence their recall of items used to measure the personality factors. Similarly, no correlation was found between any of the Big Five scores and disease duration, indicating that time elapsed after disease onset did not affect the reporting of premorbid personality scores.

The fact that ALS individuals in this study were more likely than people without ALS to have higher Agreeableness, Conscientiousness, and Extraversion, and lower Neuroticism, may have implications for the pathogenesis of the disease. Personality has a major genetic component (Vukasovic & Bratko, 2015), and it has been suggested that the same genetic factors that give rise to personality could predispose people to developing ALS (Grossman et al., 2006). Increasing numbers of studies have linked individual personality traits (in particular extraversion, which was strongly associated with ALS in the present study) with genomic loci (Lo et al., 2017). It may therefore be worthwhile examining the frequency of polymorphisms in these loci in the large genomic databases of people with and without ALS that are now available.

One possible link between personality traits and ALS is that the premorbid personality differences seen in people with ALS could lead to behaviors that are reported to be risk factors for the disease (Figure 7). Connections between personality traits and ALS risk factors could, for example, exist between extraversion‐linked smoking (Buczkowski et al., 2017), a reported risk factor for ALS (Armon, 2009); extraversion‐linked risk‐taking behavior (Levenson, 1990), which could lead to the increased numbers of head injuries reported in ALS (Schmidt, Kwee, Allen, & Oddone, 2010); conscientiousness‐linked physical exercise (Malinauskas, Dumciene, Mamkus, & Venckunas, 2014), another suggested risk factor for ALS (Beghi et al., 2010); conscientiousness‐linked low alcohol intake (Lunn, Nowson, Worsley, & Torres, 2014), which is associated with ALS (de Jong et al., 2012); conscientiousness‐linked adherence to dietary advice on increased fish intake (Lunn et al., 2014), which may be a risk factor for ALS due to mercury ingestion (Andrew et al., 2018); and either conscientiousness, openness, neuroticism, or agreeableness all of which can influence choice of occupation (Zhao & Seibert, 2006), since certain occupations have been associated with ALS (Sutedja et al., 2009). These potential links imply that some of the lifestyle habits and choices thought to be risk factors for ALS may not be risk factors per se, but rather the consequence of an underlying, substantially genetically determined, personality type.

Results of ALS risk factor studies often vary between different countries. For example, smoking is reported to be an ALS risk factor in some countries (Armon, 2009), but not in others (Pamphlett & Ward, 2012). The same is true for exercise, with variable results between countries (Lacorte et al., 2016). This puzzling feature may be related to personality, since large variations in personality, particularly in conscientiousness and neuroticism, have been described across ten world regions (Schmitt et al., 2007). This means that people in different parts of the world may be predisposed to expose themselves to dissimilar risk factors. For example, populations having higher levels of conscientiousness might be less likely to smoke and those with lower conscientiousness more likely to smoke. We do not yet have sufficient numbers of respondents from different countries to investigate the effects of nation‐specific personality types on ALS, but we plan to do so when we obtain more questionnaire respondents in this ongoing project.

The limitations of online questionnaires in the investigation of ALS risk factors have recently been summarized (Parkin Kullmann & Pamphlett, 2017). Limitations specific to the present study are as follows: (a) ALS respondents were asked to remember their personality aspects that applied to them *before* they were diagnosed. This is, however, of less concern in a disease like ALS which usually has a short course (as shown by the median disease duration of 3 years in the present study), than in disorders with long courses such as multiple sclerosis, where people would need to recall these aspects many years after diagnosis. (b) A potential limitation is that people with a particular personality type, especially those with high conscientiousness, are more likely to complete a detailed questionnaire. This would, however, apply to both ALS respondents and controls and should not therefore affect the comparative results. (c) Our ALS respondents provided a questionnaire response at only one time point in their disease course, so we are not able to determine whether premorbid personality had an effect on the rate of disease progress or the total duration of disease until death. However, others have studied the effects of post‐ALS onset psychological factors and disease course (Krampe et al., 2008; van Groenestijn, Kruitwagen‐van Reenen, Visser‐Meily, Berg, & Schroder, 2016). (d) People with ALS‐FTD are often prone to disinhibited behavior, which might be regarded as extraversion. However, only 2% of our ALS respondents reported being diagnosed with ALS‐FTD, so the inclusion of this group is unlikely to have had a significant effect on the overall results. Furthermore, it is unlikely that respondents with moderate or severe dementia would be capable of completing the detailed questionnaire. (e) Numbers of respondents in the nonclassical ALS subgroups (progressive muscular atrophy, primary lateral sclerosis, progressive bulbar palsy, and ALS/FTD) were too small, when separated by gender, to assess whether personality differences were statistically different in these individual subgroups. (f) We have no way of telling if some people with ALS, most likely due to muscle weakness, needed help from another person to complete the questionnaire. The questionnaire format was compatible with computer assistive technologies such as voice‐activated systems, but some people with bulbar weakness would not have been able to use this. We think, however, that having help to complete the questionnaire would not affect the results. (g) There is a possibility that non‐ALS controls over the age of 40 years could develop ALS later. However, ALS is a relatively uncommon disorder, with a lifetime risk of about 1:350 for men and 1:400 for women (Van Es et al., 2017), so since we had 120 male and 282 female controls, only one of our controls in total is likely to develop ALS during their lifetime, which would not affect the results.

In conclusion, people with ALS appear more likely than controls to have personality traits that can be considered to represent “niceness.” Possibilities that need to be considered are whether personality differences in ALS influence lifestyle choices (and therefore disease risk factors) and whether genetic variants influencing personality could be associated with ALS itself. To take into account the influence of personality on lifestyle choices, we recommend that personality testing be included in future investigations of risk factors for ALS.
